# Effect of an integrated intervention package of preventive chemotherapy, community-led total sanitation and health education on the prevalence of helminth and intestinal protozoa infections in Côte d’Ivoire

**DOI:** 10.1186/s13071-018-2642-x

**Published:** 2018-02-27

**Authors:** Eveline Hürlimann, Kigbafori D. Silué, Fabien Zouzou, Mamadou Ouattara, Thomas Schmidlin, Richard B. Yapi, Clarisse A. Houngbedji, Kouassi Dongo, Bernadette A. Kouadio, Siaka Koné, Bassirou Bonfoh, Eliézer K. N’Goran, Jürg Utzinger, Cinthia A. Acka-Douabélé, Giovanna Raso

**Affiliations:** 1 Swiss Tropical and Public Health Institute, P.O. Box, CH-4002 Basel, Switzerland; 2 University of Basel, P.O. Box, CH-4003 Basel, Switzerland; 3 Centre Suisse de Recherches Scientifiques en Côte d’Ivoire, 01 BP 1303, Abidjan, 01 Côte d’Ivoire; 4 Unité de Formation et de Recherche Biosciences, Université Félix Houphouët-Boigny, 22 BP 522, Abidjan, 22 Côte d’Ivoire; 5 Fairmed, Aarbergergasse 29, CH-3011 Bern, Switzerland; 6 Unité de Formation et de Recherche des Sciences de la Nature, Université Nangui Abrogoua, 02 BP 801, Abidjan, 02 Côte d’Ivoire; 7 Université Alassane Ouattara, 01 BP v 18, Bouaké, 01 Côte d’Ivoire; 8 International Millennial HeadQuarters - Bureau des Nations Unies au Burundi, United Nations Children’s Fund (UNICEF), Chaussée d’UvirWa, BP 1650, Bujumbura, Burundi

**Keywords:** Community-led total sanitation, Côte d’Ivoire, Health education, Integrated control, Intestinal protozoa, Neglected tropical diseases, Schistosomiasis, Soil-transmitted helminthiasis

## Abstract

**Background:**

Preventive chemotherapy with donated anthelminthic drugs is the cornerstone for the control of helminthiases. However, reinfection can occur rapidly in the absence of clean water and sanitation coupled with unhygienic behaviour. The purpose of this study was to assess the effect of an integrated package of interventions, consisting of preventive chemotherapy, community-led total sanitation (CLTS) and health education, on the prevalence of helminth and intestinal protozoa infections and on participants’ knowledge, attitude, practice and beliefs (KAPB) towards these diseases including water, sanitation and hygiene (WASH).

**Methods:**

A cross-sectional survey was carried out in nine communities of south-central Côte d’Ivoire to assess people’s infection with helminths and intestinal protozoa and KAPB. Subsequently, interventions were targeted to five communities, while the remaining communities served as control. The intervention encouraged latrine construction and an evaluation was done 6–7 months later to determine open defecation status of the respective communities. Anthelminthic treatment was provided to all community members. A follow-up cross-sectional survey was conducted approximately one year later, using the same procedures.

**Results:**

Overall, 810 people had complete baseline and follow-up data and were given anthelminthic treatment. The baseline prevalence of hookworm, *Schistosoma haematobium*, *Trichuris trichiura*, *Schistosoma mansoni* and *Ascaris lumbricoides* was 31.1%, 7.0%, 2.0%, 1.0% and 0.3%, respectively. Four of the five intervention communities were classified open-defecation free. For hookworm infection, we observed higher negative changes in terms of proportion of decrease (-0.10; 95% confidence interval (CI): - 0.16, -0.04) and higher egg reduction rate (64.9 *vs* 15.2%) when comparing intervention with control communities. For intestinal protozoa, prevalence reduction was higher in intervention compared to control communities (8.2 *vs* 2.6%) and WASH indicators and intervention outcomes associated with lower odds for infection at follow-up. The intervention significantly impacted on reported latrine use (before: 15.5%, after: 94.6%), open defecation in the community surroundings (before: 75.0%, after: 16.7%) and awareness for environmental contamination through open defecation (before: 20.4%, after: 52.2%).

**Conclusions:**

An integrated package of interventions consisting of preventive chemotherapy, health education and CLTS reduces the prevalence of helminth and intestinal protozoa infection. Additional studies in other social-ecological settings are warranted to confirm our findings.

**Electronic supplementary material:**

The online version of this article (10.1186/s13071-018-2642-x) contains supplementary material, which is available to authorized users.

## Background

More than half of the human population is at risk of soil-transmitted helminthiasis and schistosomiasis, over 1 billion people are infected and the global burden of these neglected tropical diseases in 2015 was 6.3 million disability-adjusted life years (DALYs) [1]. The strategy put forth by the World Health Organization (WHO) for the control of helminthiases is preventive chemotherapy that is the periodic administration of anthelminthic drugs to at-risk populations, particularly school-aged children. Albendazole and mebendazole against soil-transmitted helminthiasis and praziquantel against schistosomiasis are the drugs of choice [2]. However, reinfection may occur rapidly as long as access to clean water, adequate sanitation and hygiene behaviour have not been improved [3]. Often control efforts do not take these latter aspects sufficiently into account, despite the evidence that water supply and sanitation are key factors for prevention and sustainable control of helminthiases [4–6].

Additionally, the use of improved over unimproved sanitation and excreta disposal (use of any type of latrine/toilet *vs* open defecation) were associated with significantly lower odds of diarrhoea [7, 8]. While recent randomised controlled trials assessing the effect of water, sanitation and hygiene (WASH) on the prevalence and incidence of intestinal parasite infections and diarrhoeal diseases revealed somewhat mixed results, integrated approaches consisting of preventive chemotherapy, WASH interventions and health education were more effective [9–12] than single interventions [13]. It is important to note that measurability of protective effects from sanitation interventions depends on social-ecological contexts (e.g. resource-scarce environment *vs* area with improved facilities at baseline [14], rural *vs* urban [15]), latrine coverage [16] and how rigorously interventions were implemented (such as elimination of open defecation *vs* increase of latrine ownership) [17]. Prior research underscores the importance of integrated approaches for effective control of helminthiases and diarrhoeal diseases [18–20]. Yet, there is a need for well-designed studies on water and sanitation interventions, health education and preventive chemotherapy against neglected tropical diseases.

Community-led total sanitation (CLTS) is a participatory approach that aims to achieve and sustain an open defecation free (ODF) status at the community level [21]. This approach has the potential to reduce the incidence of helminth and intestinal protozoa infections and, when implemented alongside preventive chemotherapy, might provide a sustainable way to control neglected tropical diseases. However, to our knowledge, the effect on reinfection patterns with helminth and intestinal protozoa infections has yet to be determined. CLTS employs participatory rural appraisal and is based on the belief that the learning effect is much higher if the knowledge is acquired through self-experience and self-reflexion [22]. The approach facilitates the critical analysis by the community of their own sanitation-profile, inappropriate defecation practices and the consequences, leading to collective action to become ODF [23]. CLTS is a community-based and -led strategy that triggers community empowerment via feelings like shame and disgust induced through observation of the defecation situation in a specific setting and its environment, which is much harder to address by health education [24, 25].

The purpose of this study was to assess the effect of an integrated approach that combines preventive chemotherapy with CLTS and health education, on reinfection patterns with helminths and intestinal protozoa and on knowledge, attitude, practice and beliefs (KAPB) of hygiene in the Taabo health and demographic surveillance system (HDSS) in south-central Côte d’Ivoire.

## Methods

### Study area and population

The study was carried out in the Taabo HDSS, located in south-central Côte d’Ivoire [26]. The study area and the baseline situation with regard to hygiene and sanitation conditions have been described elsewhere [27]. In brief, open defecation is common. In addition to agricultural activities, local populations raise animals such as pigs, goats, sheep and chicken in their immediate living environment that may also influence disease transmission (e.g. pigs consume human faeces). Preventive chemotherapy was implemented in 2008, 2009 and 2010 using albendazole, praziquantel and ivermectin for the control of soil-transmitted helminthiasis, schistosomiasis, onchocerciasis and lymphatic filariasis.

The study was implemented in two villages (Katchénou and Sahoua), and seven hamlets belonging to different villages, Yobouékro (village Sahoua), Ouattafouékro and Kouadio Kouamékro (Ahondo), Boussoukro (Tokohiri), Amani Kouadiokro (Sokrogbo) and Bêh N’Guessankro and Allah Thérèsekro (Léléblé). These villages and hamlets were selected because of their characteristics that are in favour of meaningful and successful implementation of CLTS, namely (i) small population sizes; (ii) clear signs of practiced open defecation; (iii) inhabitants that have the potential to become natural leaders; and (iv) relatively homogeneous population structure in terms of culture and socioeconomic status. Communities were randomly assigned to the intervention and control group taking into account for matching characteristics such as population size, hygiene status, village affiliation and geographic position. All residents of the villages and hamlets were invited to participate. During the study period a non-governmental organisation (NGO) visited Amani Kouadiokro and carried out CLTS. Given that for our study this hamlet was a control site, we considered this location retrospectively to be within the intervention group for analysis. Although this community had not received the additional health education package it qualified as intervention community in light of rapidly achieved ODF status that is the ultimate goal of the intervention and to be related with all outcomes assessed at follow-up.

### Study design

**Fig. 1:**
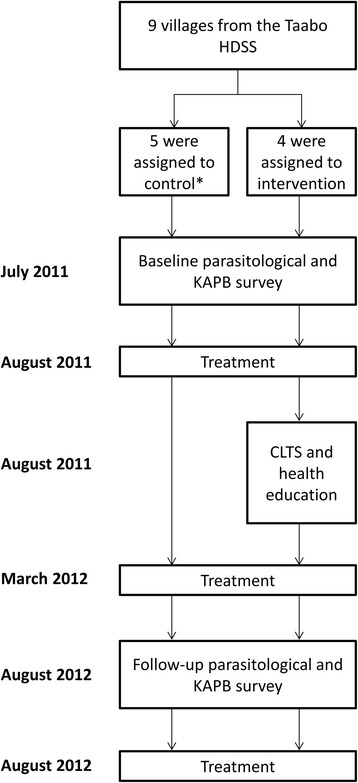
Study design. *Amani Kouadiokro was retrospectively assigned to the intervention group since CLTS was undertaken by a local non-governmental organisation in this community

Figure 1 shows the study design. In July 2011, shortly before the annual round of preventive chemotherapy, a cross-sectional survey was conducted to determine peoples’ infection status with helminths and intestinal protozoa and to assess their KAPB regarding helminthiases and hygiene [27]. Interventions (i.e. CLTS and health education) were implemented subsequently, including a second round of preventive chemotherapy. Approximately 1 year after baseline, a follow-up survey was conducted, using the same field and laboratory procedures.

### Parasitological survey

Our research team visited all villages and hamlets to obtain local approval and to explain the parasitological survey and questionnaire interviews. The day before the survey, field enumerators from the Taabo HDSS and community health workers distributed plastic containers (125 ml) to all households for stool and urine collection. Participants were invited to submit early morning stool and urine samples on the following day to a communal central place in the village/hamlet. For parasitological examinations, faecal and urine samples were transferred to a mobile field laboratory in Léléblé or Sokrogbo, or the laboratory of the hospital in Taabo-Cité depending on the sampled localities’ proximity to the respective laboratories.

Stool and urine samples were processed and examined by experienced laboratory technicians on the day of collection. In brief, stool samples were subjected to duplicate Kato-Katz thick smears, using 41.7 mg plastic templates [28]. The number of eggs of *Schistosoma mansoni*, *Ascaris lumbricoides*, *Trichuris trichiura* and hookworm were counted and recorded separately. Egg counts were multiplied by a factor of 24 to estimate the number of eggs per gram of stool (epg) [29]. Urine samples were subjected to a filtration method. Ten ml of vigorously shaken urine was passed through a Nytrel filter (Sefar AG; Heiden, Switzerland; diameter: 13 mm, mesh size: 20 μm). Individual filters were placed on a microscope slide, a drop of Lugol’s iodine added and then examined under a microscope. The number of *S. haematobium* eggs were counted and expressed as eggs per 10 ml of urine [30]. For quality control, 10% of the samples were re-examined by a senior laboratory technician and discrepancies discussed until accordance was reached.

Additionally, from each stool sample 1–2 g was processed using an ether-concentration method. The sodium acetate-acetic acid-formalin (SAF)-fixed stool samples were forwarded to a laboratory at the Centre Suisse de Recherches Scientifiques en Côte d’Ivoire (CSRS; Abidjan, Côte d’Ivoire) for examination of intestinal protozoa infections. Standard protocols were followed and intestinal protozoa (*Blastocystis* sp., *Chilomastix mesnili*, *Entamoeba coli*, *Entamoeba hartmanni*, *Entamoeba histolytica/E. dispar*, *Endolimax nana*, *Giardia intestinalis* and *Iodamoeba bütschlii*) recorded semi-quantitatively [31].

### Questionnaire survey

For the interviews with KAPB questionnaires, households were visited by a researcher accompanied by a trained field enumerator from the Taabo HDSS who speaks the local language. The questionnaire was addressed to the household head or a present adult household member in case of absence of the earlier. The questionnaire has been presented in detail elsewhere [27]. In brief, the questionnaires consisted of basic questions pertaining to demographic factors, socioeconomic indicators, sanitation, hygiene and defecation behaviour, opinions, taboos and beliefs, and concepts on intestinal parasitic infections.

### CLTS and health education

**Fig. 2:**
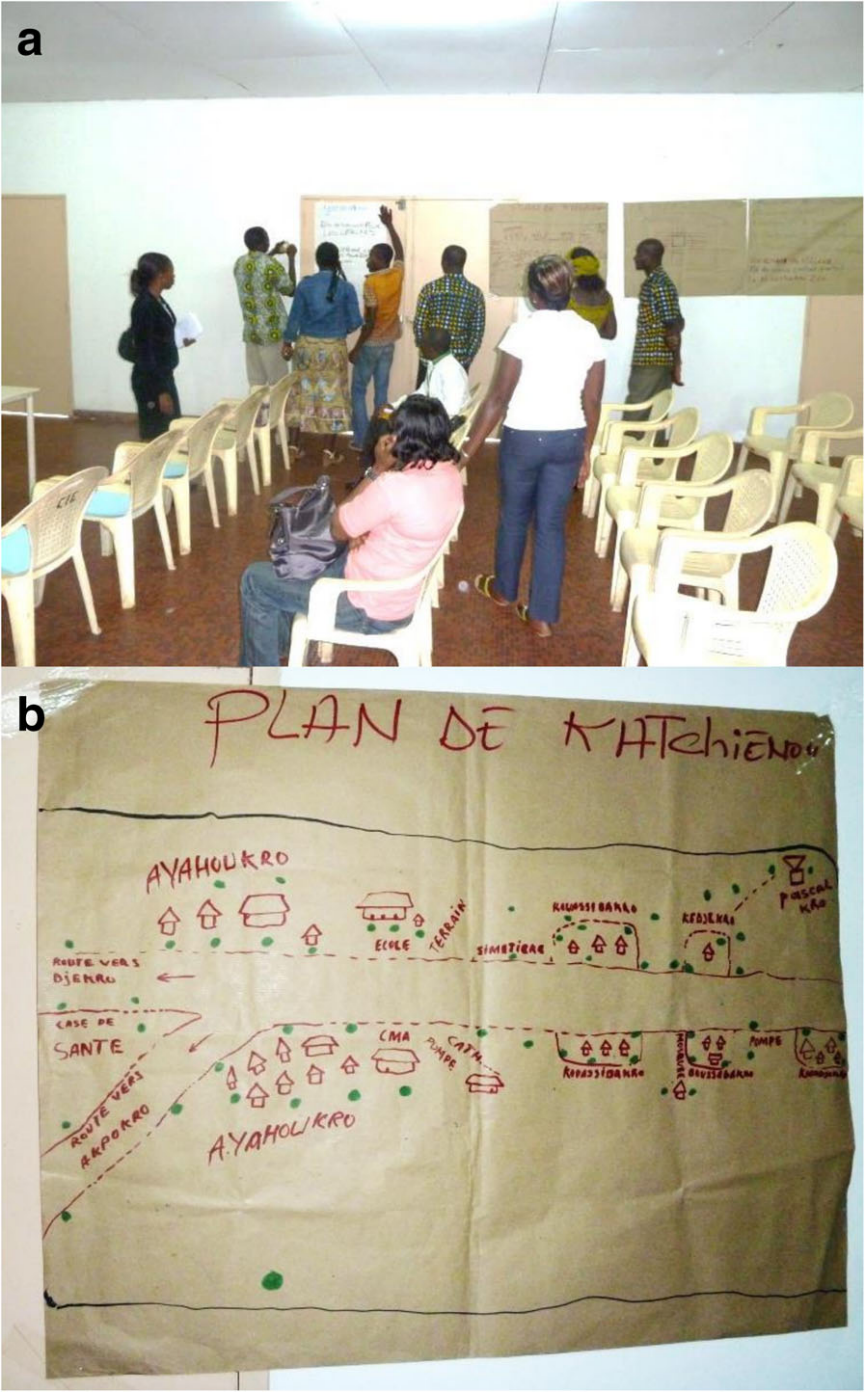
Community members presenting their action plans for CLST (**a**), including a map of their community with indicated defecation sites (**b**) during a workshop held at Taabo-Cité

The CLTS intervention was carried out according to Kar & Chambers [21]. Pre-triggering was integrated in the information visit to communities about the upcoming surveys and further enhanced during the KAPB survey, while information was collected and defecation sites located. Subsequently, a workshop was organised to train personnel from the Taabo HDSS, local health authorities, members of the mayor’s office and other stakeholders from the sub-prefecture of Taabo with regard to the CLTS approach. At this event, experienced CLTS facilitators from different institutions (CSRS, UNICEF and the Ministry of Health and Public Hygiene) presented the CLTS approach and the different tactics of implementing CLTS and leading the community to the ignition moment. Teams were then built, each including a lead-facilitator, co-facilitators, content and process recorders and one person responsible for organisational issues and communities that were chosen for the intervention visited. The visit (triggering) lasted half a day and went as follows. First, a meeting with the community leaders was held, encouraging all community members to participate. Secondly, several activities were done with the community members, including mapping of community and defecation areas, identifying dirtiest neighbourhoods on the map, calculation of “caca” and medical expenses due to diarrhoea, transect walk through the open defecation areas (“walk of shame”), triggering disgust of faecal contamination of food, water or hands [showing, for example, how flies fly from faeces to food (“caca-to-food”)]. The latter activity is where the ignition moment took place in general, namely when people realise that they are eating each other’s faeces. In a next step, the community was motivated to plan their decisions, actions and time schedule until when the community will be ODF. Third, a closing ceremony was organised by the team where active and motivated community members identified during the intervention (natural leaders) were invited to present in front of all invited local authorities their plan of action (Fig. 2).

After triggering, the action of the community was supported and followed-up by regular visits every 2 weeks by the facilitators. During these follow-ups, the facilitators visited the communities to assist and monitor the construction of latrines. People were constantly encouraged to help each other to build latrines or share latrines until every household had its own latrine [21, 22, 32]. ODF status was evaluated 10 and 28 weeks after triggering through active search for remaining open defecation sites during unannounced visits in the community and its surroundings.

The whole process of CLTS was supported by specific health education sessions using participatory rural appraisal tools. This approach included three major aspects, namely (i) evaluating existing knowledge; (ii) health education provided to the whole community and to separate groups (e.g. men, women, children and health committees) through focus group discussions led by a social scientist; and (iii) set up of an action plan for continued provision of health education. The facilitators discussed the health risks associated to inappropriate hygiene, open defecation and other risk behaviour with groups of community members. Risk behaviour for helminth and intestinal protozoa infections, transmission cycles and preventive measures were described using visual tools such as booklets, posters and pictures. These tools were handed over to community health committees in charge of promoting good hygiene behaviour in the community that were built as part of the intervention.

### Statistical analysis

Data were double-entered and cross-checked using EpiInfo version 3.5.1 (Centers for Disease Control and Prevention, Atlanta, GA, USA) and analysed using STATA version 10.0 (Stata Corp., College Station, TX, USA). Only participants with complete datasets (i.e. those with duplicate Kato-Katz thick smears, one SAF-fixed faecal sample and one urine filtration) and confirmed administration of albendazole treatment were included in the final analyses. Participants were stratified into five age groups (i.e. < 6; 6–15; 16–29; 30–45; and > 45 years). Logistic regression models for infection status, behaviour and awareness with regard to defecation and hygiene were used to assess the association with the intervention at baseline and the 1-year follow-up. Prevalence reduction was defined as (prevalence at baseline – prevalence at follow-up). Negative binomial regression models were used to assess the association between faecal egg counts of single helminth species infections and the intervention. Egg count reduction, expressed as egg reduction rate (ERR), was calculated as [1 – (geometric mean epg at follow-up/geometric mean epg at baseline)] multiplied by a factor of 100. Significant changes between baseline and follow-up frequencies for infection status, behaviour and awareness with regard to defecation and hygiene within the respective group were analysed using the McNemar’s test. To assess differences over time in infection status between control and intervention communities, taking into account for different baseline prevalence levels, we compared (i) total proportion of change; (ii) proportion of decrease; and (iii) proportion of increase [33, 34]. Differences in proportion of change were considered significant in case of non-overlapping 95% confidence intervals (CIs) based on the following formula:

$$ SE\left({p}_{g1}-{p}_{g2}\right)=\sqrt{\left[\frac{p_{g1}\left(1-{p}_{g1}\right)}{N_{g1}}\right]+\left[\frac{p_{g2}\left(1-{p}_{g2}\right)}{N_{g\;2}}\right]} $$

where *SE* is the standard error, *p*_g1_ is the proportion of change in group 1, *p*_g2_ is the proportion of change in group 2, *N*_g1_ is the number of subjects in group 1, and *N*_g2_ is the number of subjects in group 2.

Univariate and multivariable relationship analysis to assess guiding risk factors for the follow-up infection status was done using logistic regression modelling, including a random effect coefficient on community level. Results of this analysis were presented as crude and adjusted odds ratios (ORs). The latter were adjusted for sociodemographic indicators, namely sex, age group, socioeconomic status and ethnic origin.

## Results

### Participants

There were 3420 people living in the nine selected communities according to the Taabo HDSS census database and 3289 were present and participated during the baseline survey in 2011. A total of 2413 provided stool samples, 2294 submitted urine samples and questionnaire data were available from 3152 people. Overall, 1894 people had complete parasitological and questionnaire data. Results from the baseline survey have been presented elsewhere [27]. For the 1-year follow-up survey done in 2012, a total of 3290 people were present, of which 2064 provided stool and 1902 urine samples, while questionnaire data were obtained from 3182 people. Complete parasitological and questionnaire data were available from 1481 people.

A total of 810 individuals had complete parasitological and questionnaire data from both the baseline and 1-year follow-up surveys and had received anthelminthic treatment after the second ODF evaluation in March 2012. Age and sex characteristics of this final cohort are presented in Additional file 1: Table S1.

### Community response following the interventions

**Fig. 3:**
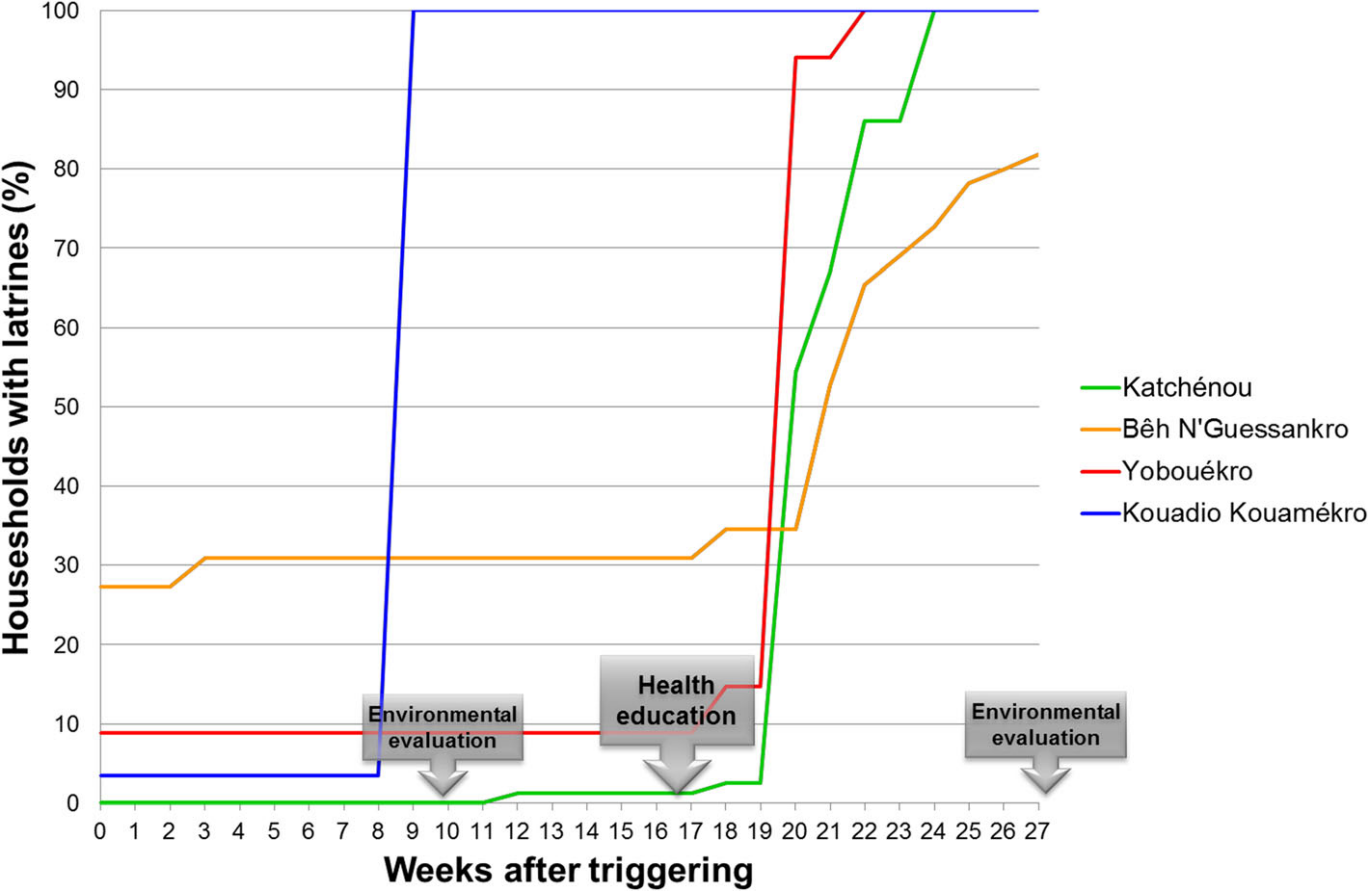
Dynamics of latrine construction after triggering of CLTS and as response to a health education intervention

Table 1 summarises information on latrines owned by households before and after the intervention. At baseline, coverage of latrine ownership at the unit of the community varied from 0% (i.e. Katchéneou) to 50% (i.e. Allah Thérèsekro). Following two rounds of evaluations, four out of five intervention communities reached a 100% household coverage with latrines and were verified ODF. The remaining intervention community reached a latrine coverage of more than 80% but continued open defecation was still recorded. The proportion of latrine ownership at follow-up in control communities remained comparable with the baseline proportion. Dynamics of latrine construction among intervention communities are shown in Fig. 3. Kouadio Kouamékro and Amani Kouadiokro (not shown in figure) already reached 100% latrine coverage and ODF status after the first environmental evaluation 10 weeks after triggering. The other three intervention communities only started to extensively construct latrines following a specific health education package.

**Table 1:** Characteristics of the selected villages/hamlets and results from the evaluation prior to certification

Type of intervention	Community	Registered inhabitants (*n* = 3420)	Registered households (*n* = 487)	Households with latrines at baseline (%)	Households with latrines at follow-up (%)	ODF status^c^
Preventive chemotherapy	Allah Thérèsekro	320	56	28 (50.0)	34 (60.7)	
Preventive chemotherapy^a^	Boussoukro	294	48	12 (25.0)	15 (31.3)	
Preventive chemotherapy^a^	Ouattafouékro	157	29	1 (3.4)	1 (3.4)	
Preventive chemotherapy	Sahoua	872	118	25 (21.2)	31 (26.3)	
Preventive chemotherapy + CLTS^b^	Amani Kouadiokro	253	24	0 (0)	24 (100)	Yes
Preventive chemotherapy + CLTS + health education	Bêh N’Guessankro	393	55	15 (27.3)	45 (81.82)	No
Preventive chemotherapy + CLTS + health education	Katchénou	713	91	0 (0)	91 (100)	Yes
Preventive chemotherapy + CLTS + health education	Kouadio Kouamékro	171	29	1 (3.4)	29 (100)	Yes
Preventive chemotherapy + CLTS + health education	Yobouékro	247	37	3 (8.1)	37 (100)	Yes

*Abbreviations*: *CLTS* community-led total sanitation, *ODF* open defecation free

^a^Boussoukro and Ouattafouékro are two hamlets situated 1 km apart from each other. For randomisation these two hamlets were considered as one unit

^b^Amani Kouadiokro, a hamlet at the border of the sub-prefecture, was initially attributed to the control group during randomisation, however, an NGO intervening at the adjacent sub-prefecture erroneously visited the hamlet and carried out a CLTS intervention. Thus, this community was attributed to the intervention group for analysis of parasitological data

^c^Two evaluations took place. The first evaluation was implemented on 26–27 November 2011, the second took place on 19–25 March 2012. Kouadio Kouamékro and Amani Kouadiokro were certified ODF following the first evaluation, whereas Katchénou and Yobouékro were certified ODF following the second evaluation

### Impact of the intervention on prevalence and intensity of infection

The prevalence of helminth and intestinal protozoa infection at baseline and follow-up is shown in Table 2. Baseline helminth infections were not comparable between control and intervention communities. *S. haematobium* infection, the predominant *Schistosoma* species, was mainly found in Sahoua, belonging to the control group. This is reflected in a significantly reduced OR for schistosomiasis in intervention communities (OR = 0.05, *P* <  0.001). Intervention communities were more affected by soil-transmitted helminth infection (mainly hookworm) than control communities at baseline (38.0 *vs* 25.7%, OR = 1.77, *P* <  0.001). However, at follow-up helminth infection prevalence was low among both control and intervention communities with 7.0% and 11.8% for soil-transmitted helminths and 5.7% and 1.4%, respectively, for *Schistosoma* spp. infections. For intestinal protozoa infections that were equally frequent in both groups at baseline, a significant decline from 72.0% to 63.8% was observed in intervention communities whereas control communities still showed similar frequencies as at baseline (73.0% and 70.4%).

**Table 2:** Comparison of infection prevalences among 810 participants, stratified by control and intervention communities during baseline and follow-up surveys. Logistic regressions were used to derive odds ratios for comparison of prevalences of control and intervention communities at baseline and follow-up. The prevalence reduction was calculated as baseline prevalence minus follow-up prevalence for control and intervention communities separately

Parasite	Group	Baseline prevalence (%)	OR (95% CI)	*P*-value	Follow-up prevalence (%)	OR (95% CI)	*P*-value	Prevalence reduction (%)
*Schistosoma* spp.	Control	15.58	1		5.71	1		9.87^*^
	Intervention	0.94	0.05 (0.02–0.14)	< 0.001	1.41	0.24 (0.10–0.59)	0.002	-0.47
*Schistosoma mansoni*	Control	1.04	1		1.04	1		0.00
	Intervention	0.94	0.90 (0.22–3.64)	0.888	0.71	0.68 (0.15–3.04)	0.611	0.23
*Schistosoma haematobium*	Control	14.81	na		4.68	1		10.13^*^
	Intervention	0	na		0.71	0.15 (0.04–0.50)	0.002	-0.71
Soil-transmitted helminths	Control	25.71	1		7.01	1		18.70^*^
	Intervention	37.97	1.77 (1.32–2.40)	< 0.001	11.76	1.77 (1.08–2.89)	0.023	26.21^*^
Hookworm	Control	25.19	1		7.01	1		18.18^*^
	Intervention	36.47	1.70 (1.26–2.31)	0.001	10.59	1.57 (0.95–2.58)	0.076	25.88^*^
*Ascaris lumbricoides*	Control	0	na		0	na		0.00
	Intervention	0.71	na		0.24	na		0.47
*Trichuris trichiura*	Control	1.30	1		0	na		1.30^*^
	Intervention	2.59	2.02 (0.70–5.87)	0.196	1.41	na		1.18
Intestinal helminths^a^	Control	26.23	1		7.79	1		18.44^*^
	Intervention	38.44	1.77 (1.31–2.38)	< 0.001	12.47	1.69 (1.05–2.70)	0.030	25.97^*^
Intestinal protozoa	Control	72.99	1		70.39	1		2.60
	Intervention	72.00	0.95 (0.70–1.30)	0.753	63.76	0.74 (0.55–0.99)	0.046	8.24^*^
*Entamoeba histolytica/E. dispar*	Control	9.87	1		9.61	1		0.26
	Intervention	17.65	1.96 (1.29–2.97)	0.002	10.59	1.11 (0.70–1.76)	0.645	7.06^*^
*Entamoeba coli*	Control	44.16	1		40.52	1		3.64
	Intervention	40.47	0.86 (0.65–1.14)	0.289	39.53	0.96 (0.72–1.27)	0.774	0.94
*Entamoeba harmanni*	Control	1.30	1		4.94	1		-3.64^*^
	Intervention	1.65	1.27 (0.40–4.04)	0.683	1.18	0.23 (0.05–0.62)	0.004	0.47
*Endolimax nana*	Control	26.23	1		29.61	1		-3.38
	Intervention	29.65	1.18 (0.87–1.61)	0.280	24.00	0.75 (0.55–1.02)	0.072	5.65
*Iodamoeba bütschlii*	Control	15.58	1		8.05	1		7.53^*^
	Intervention	18.35	1.22 (0.84–1.76)	0.296	7.76	0.96 (0.58–1.60)	0.880	10.59^*^
*Giardia intestinalis*	Control	16.36	1		14.03	1		2.33
	Intervention	16.94	1.04 (0.72–1.51)	0.826	12.24	0.86 (0.57–1.29)	0.451	4.70^*^
*Chilomastix mesnili*	Control	4.42	1		4.94	1		-0.52
	Intervention	5.18	1.18 (0.62–2.26)	0.614	5.18	1.05 (0.56–1.97)	0.876	0.00
*Blastocystis* sp.	Control	34.03	1		29.09	1		4.94
	Intervention	35.53	1.07 (0.80–1.43)	0.654	23.53	0.75 (0.55–1.03)	0.073	12.0^*^

*Abbreviations: OR* odds ratio, *CI* confidence interval, *na* not applicable

^a^Includes soil-transmitted helminth and *S. mansoni* infections

^*^Significant change between 2011 and 2012 according to McNemar’s test (*P* <  0.05)

Helminth infection intensities at baseline and follow-up are shown in Additional file 2: Table S2. ERRs for hookworm were considerably higher for intervention than control communities (64.9 *vs* 15.2%) due to higher baseline egg counts in the intervention communities. There was no significant difference in hookworm egg counts between intervention and control group after treatment.

Table 3 shows significant differences in the proportion of change in helminth and intestinal protozoa infections between intervention and control communities. Intervention communities showed a significantly higher proportion of decrease for soil-transmitted helminth infection, hookworm and *E. histolytica*/*E. dispar* infection, while control communities showed significantly higher changes in proportion of increase for *E. nana* and *Blastocystis* sp. infections.

**Table 3:** Significant differences in proportion of change in helminth and intestinal protozoa infections between intervention and control group. Positive differences in proportion of change indicate higher changes in control communities, while negative differences attribute a higher proportion of change in intervention communities

Parasite	Group	Change	Proportion of change	Difference in proportion (SE)	95% CI
Soil-transmitted helminth infection	Control	All	0.23	-0.12 (0.03)	-0.18, -0.06^*^
	Intervention		0.35		
	Control	Decrease	0.21	-0.10 (0.03)	-0.16, -0.04^*^
	Intervention		0.31		
	Control	Increase	0.02	-0.02 (0.01)	-0.05, 0.00
	Intervention		0.04		
Hookworm	Control	All	0.23	-0.10 (0.03)	-0.16, -0.04^*^
	Intervention		0.33		
	Control	Decrease	0.21	-0.09 (0.03)	-0.15, -0.03^*^
	Intervention		0.29		
	Control	Increase	0.02	-0.01 (0.01)	-0.04, 0.01
	Intervention		0.04		
*E. histolytica*/*E. dispar*	Control	All	0.16	-0.08 (0.03)	-0.14, -0.03^*^
	Intervention		0.24		
	Control	Decrease	0.08	-0.07 (0.02)	-0.12, -0.03^*^
	Intervention		0.16		
	Control	Increase	0.08	-0.01 (0.02)	-0.04, 0.03
	Intervention		0.09		
*E. nana*	Control	All	0.41	0.05 (0.03)	-0.02, 0.11
	Intervention		0.37		
	Control	Decrease	0.19	-0.02 (0.03)	-0.08, 0.03
	Intervention		0.21		
	Control	Increase	0.22	0.07 (0.03)	0.01, 0.12^*^
	Intervention		0.16		
*Blastocystis* sp*.*	Control	All	0.44	0.04 (0.03)	-0.03, 0.11
	Intervention		0.40		
	Control	Decrease	0.25	-0.01 (0.03)	-0.07, 0.05
	Intervention		0.26		
	Control	Increase	0.20	0.06 (0.03)	0.00, 0.11^*^
	Intervention		0.14		

*Abbreviations*: *SE* standard error, *CI* confidence interval

^*^Significant difference in proportion of change between control and intervention group with a non-overlapping 95% confidence interval

### Risk factors guiding infection status at follow-up

**Fig. 4:**
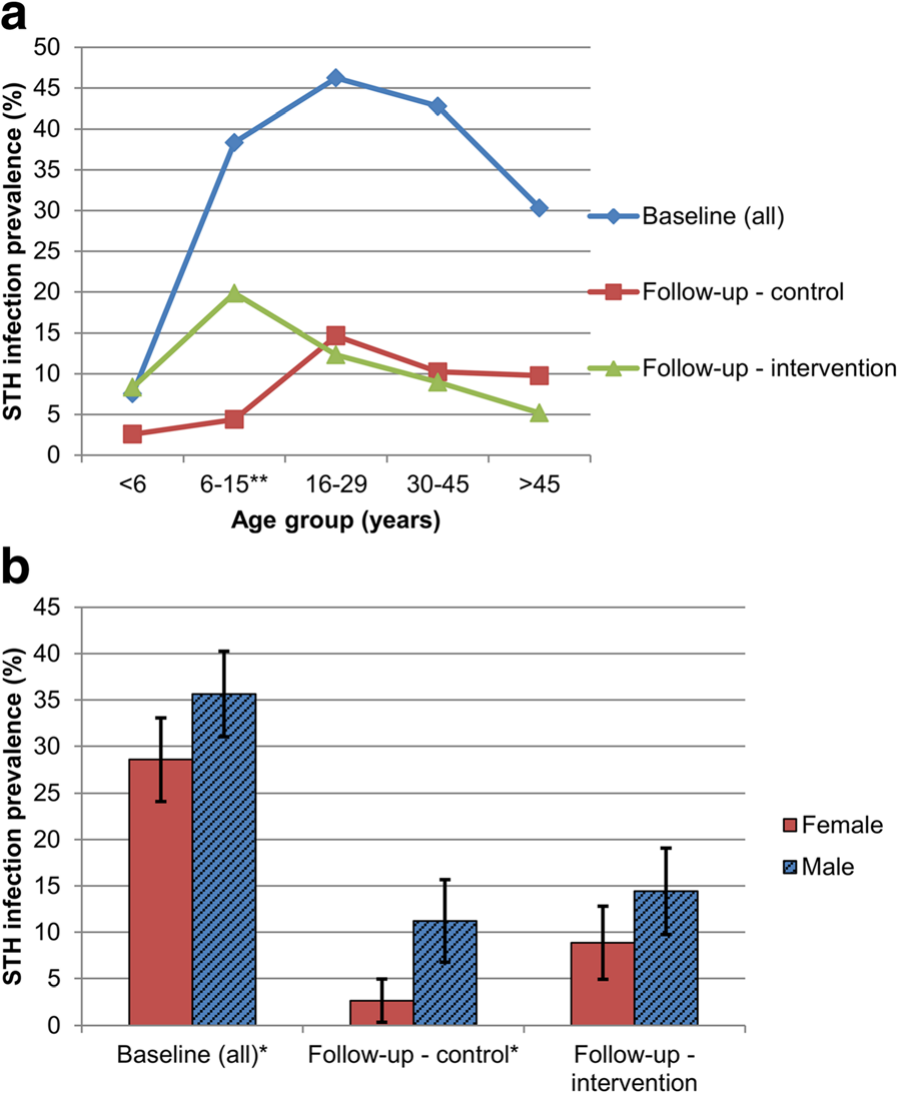
Baseline infection and reinfection patterns of soil-transmitted helminth infections in control and intervention communities by age group (**a**) and sex (**b**). **Statistically significant difference in follow-up helminth prevalence between intervention and control group for this age group (*P* <  0.001); *statistically significant difference (*P* <  0.05)

Figure 4 shows reinfection patterns with STHs in intervention and control communities, stratified by age group and sex. While soil-transmitted helminth reinfection patterns by age group in control communities followed baseline patterns with highest infection rates among adolescent and young adults (16–29 years), a peak shift towards school-aged children (6–15 years) was observed in intervention communities. In intervention communities 19.8% of school-aged children were found soil-transmitted helminth-positive compared to 4.4% in control communities (*P* <  0.001). In the former, almost half of all new infections (23/50, 46%), were diagnosed in school-aged children. Similar to age, reinfection patterns in control communities showed continued sex differences, as already observed at baseline with significantly higher rates of males infected in 2012 than females (11.2 *vs* 2.7%, *P* = 0.001), while no significant sex differences in infection status in 2012 was observed among intervention communities (14.4 *vs* 8.9%, *P* = 0.076).

Multivariable regression modelling adjusted for age, sex, socioeconomic status and ethnic origin showed no significant relationship between specific WASH indicators (e.g. toilet ownership and use) and intervention indicators (i.e. ODF status and group) with the 1-year follow-up soil-transmitted helminth infection status (Table 4). Sociodemographic and socioeconomic factors, such as male gender (OR = 2.63, *P* <  0.001) and age groups 6–15 years (OR = 2.46, *P* = 0.017) or 16–29 years (OR = 3.08, *P* = 0.010), were positively associated with reinfection with soil-transmitted helminths, whereas individuals from households with higher socioeconomic status showed significantly lower adjusted ORs (OR = 0.80, *P* = 0.007). Stratification into control and intervention communities revealed that socioeconomic factors associated with soil-transmitted helminth infection in 2012 diverged. In intervention communities, socioeconomic status (OR = 0.77, *P* = 0.015) and age group 6–15 years (OR = 3.00, *P* = 0.010) were still significantly related to reinfection. In control communities, in contrast, male gender (OR = 5.90, *P* = 0.001) and age groups 16–29 years (OR = 9.03, *P* = 0.012) and 30–45 years (OR = 5.66, *P* = 0.033) still had higher odds for soil-transmitted helminth infection in 2012.

**Table 4:** Potential risk and preventive factors guiding reinfection with soil-transmitted helminths and intestinal protozoa in 2012 from multivariable logistic regression modelling

Explanatory factor	All communities (*n* = 810)		Control group (*n* = 385)		Intervention group (*n* = 425)	
	Crude OR	Adjusted OR	Crude OR	Adjusted OR	Crude OR	Adjusted OR
	(95% CI)	(95% CI)^a^	(95% CI)	(95% CI)^a^	(95% CI)	(95% CI)^a^
*Soil-transmitted helminth infection*						
Sociodemographic indicators						
Sex (male)	2.35 (1.40–3.93)**	2.63 (1.55–4.44)**	4.79 (1.76–13.02)**	5.90 (2.11–16.50)**	1.67 (0.90–3.11)	1.72 (0.91–3.24)
Age group (6–15 years)	2.22 (1.07–4.63)*	2.46 (1.18–5.14)*	1.84 (0.36–9.40)	2.14 (0.41–11.09)	2.69 (1.17–6.15)*	3.00 (1.30–6.94)*
Age group (16–29 years)	2.33 (1.00–5.41)*	3.08 (1.30–7.28)*	6.33 (1.20–33.41)*	9.03 (1.63–50.07)*	1.53 (0.55–4.22)	1.79 (0.64–4.98)
Age group (30–45 years)	1.70 (0.76–3.81)	1.95 (0.87–4.40)	4.15 (0.86–19.98)	5.66 (1.15–28.00)*	1.05 (0.37–2.98)	1.18 (0.42–3.36)
Age group (> 45 years)	1.27 (0.47–3.42)	1.39 (0.51–3.76)	4.24 (0.73–24.52)	4.29 (0.72–25.51)	0.63 (0.16–2.46)	0.71 (0.18–2.75)
Socioeconomic index (score)	0.84 (0.71–0.98)*	0.80 (0.68–0.94)**	0.83 (0.65–1.07)	0.82 (0.66–1.02)	0.80 (0.63–1.01)	0.77 (0.62–0.95)*
Ethnic origin (autochthonous)^b^	1.34 (0.62–2.94)	1.76 (0.79–3.95)	2.68 (0.67–10.76)	1.92 (0.65–5.64)	0.92 (0.35–2.39)	1.29 (0.51–3.28)
Religion (Muslim)	0.69 (0.29–1.63)	0.61 (0.29–1.28)	0.44 (0.16–1.24)	0.50 (0.17–1.52)	1.06 (0.33–3.36)	0.84 (0.27–2.65)
Religion (animist)	1.31 (0.76–2.25)	1.42 (0.84–2.41)	1.31 (0.52–3.33)	0.86 (0.31–2.36)	1.32 (0.69–2.52)	1.54 (0.81–2.92)
Religion (other)	0.38 (0.05–3.09)	0.33 (0.04–2.51)	0.31 (0.04–2.52)	0.33 (0.04–2.78)	na	na
WASH/intervention indicators^c^						
Group (intervention)	1.62 (0.72–3.65)	1.55 (0.93–2.59)	na	na	na	na
ODF status (odf)	1.47 (0.64–3.37)	1.48 (0.90–2.42)	na	na	na	na
Toilet ownership (new owner)	1.62 (0.77–3.40)	1.53 (0.88–2.68)	0.81 (0.10–6.61)	1.04 (0.12–9.26)	1.88 (0.48–7.31)	1.37 (0.35–5.34)
Toilet ownership (still owner)	0.79 (0.33–1.85)	1.12 (0.48–2.65)	0.87 (0.34–2.18)	1.20 (0.45–3.19)	0.46 (0.04–4.92)	0.48 (0.04–5.38)
Possession of functional latrine in 2012	1.25 (0.66–2.36)	1.52 (0.88–2.61)	0.93 (0.39–2.23)	1.23 (0.49–3.09)	1.55 (0.42–5.80)	1.26 (0.35–4.52)
Toilet use in 2012	1.04 (0.52–2.09)	1.44 (0.82–2.56)	0.59 (0.24–1.47)	0.87 (0.36–2.12)	na	na
Strict toilet use in 2012	1.25 (0.70–2.22)	1.40 (0.84–2.34)	0.72 (0.29–1.82)	0.89 (0.34–2.35)	1.82 (0.68–4.85)	1.20 (0.43–3.30)
*Intestinal protozoa infection*						
Sociodemographic indicators						
Sex (male)	0.78 (0.58–1.05)	0.80 (0.60–1.08)	0.67 (0.43–1.04)	0.69 (0.44–1.08)	0.90 (0.61–1.34)	0.94 (0.63–1.41)
Age group (6–15 years)	1.06 (0.71–1.58)	1.10 (0.74–1.65)	0.95 (0.52–1.74)	0.97 (0.52–1.79)	1.09 (0.64–1.85)	1.16 (0.67–2.00)
Age group (16–29 years)	1.23 (0.74–2.05)	1.22 (0.73–2.03)	0.73 (0.33–1.61)	0.68 (0.30–1.52)	1.73 (0.90–3.34)	1.81 (0.93–3.53)
Age group (30–45 years)	1.58 (1.00–2.49)	1.58 (0.99–2.51)	1.42 (0.71–2.85)	1.35 (0.67–2.73)	1.61 (0.87–2.97)	1.65 (0.89–3.06)
Age group (> 45 years)	1.12 (0.67–1.88)	1.14 (0.68–1.92)	0.81 (0.36–1.81)	0.81 (0.36–1.85)	1.36 (0.70–2.63)	1.41 (0.72–2.76)
Socioeconomic index (score)	1.00 (0.92–1.08)	1.02 (0.94–1.10)	0.96 (0.87–1.04)	1.00 (0.91–1.11)	1.04 (0.91–1.18)	1.04 (0.91–1.18)
Origin (autochthonous)^b^	1.51 (1.03–2.22)*	1.54 (1.04–2.28)*	1.50 (0.96–2.34)	1.50 (0.91–2.48)	1.63 (0.93–2.88)	1.70 (0.96–3.04)
Religion (Muslim)	0.62 (0.40–0.97)*	0.61 (0.39–0.96)*	0.61 (0.36–1.04)	0.63 (0.35–1.11)	0.48 (0.25–0.94)*	0.46 (0.23–0.91)*
Religion (animist)	1.01 (0.70–1.45)	1.01 (0.70–1.46)	0.73 (0.39–1.35)	0.74 (0.39–1.41)	1.17 (0.76–1.81)	1.20 (0.77–1.86)
Religion (other)	1.43 (0.57–3.60)	1.45 (0.58–3.67)	1.27 (0.50–3.21)	1.35 (0.53–3.44)	na	na
WASH/intervention indicators^c^						
Group (intervention)	0.74 (0.55–0.99)*	0.68 (0.49–0.93)*	na	na	na	na
ODF status (odf)	0.73 (0.55–0.98)*	0.69 (0.50–0.93)*	na	na	na	na
Toilet ownership (new owner)	0.65 (0.47–0.91)*	0.63 (0.44–0.88)**	0.69 (0.25–1.95)	0.79 (0.27–2.31)	0.80 (0.35–1.81)	0.76 (0.33–1.77)
Toilet ownership (still owner)	0.87 (0.55–1.37)	0.88 (0.54–1.42)	0.79 (0.49–1.28)	0.84 (0.50–1.42)	1.78 (0.46–6.91)	1.58 (0.39–6.33)
Toilet ownership (no longer owner)	0.58 (0.16–2.12)	0.61 (0.17–2.25)	0.57 (0.16–2.08)	0.61 (0.17–2.27)	na	na
Possession of functional latrine in 2012	0.72 (0.52–0.98)*	0.69 (0.50–0.95)*	0.79 (0.50–1.25)	0.85 (0.52–1.38)	0.82 (0.36–1.87)	0.78 (0.33–1.81)
Toilet use in 2012	0.67 (0.48–0.94)*	0.67 (0.47–0.94)*	0.79 (0.51–1.23)	0.86 (0.54–1.38)	0.19 (0.02–1.54)	0.21 (0.03–1.67)
Strict toilet use in 2012	0.73 (0.54–0.99)*	0.73 (0.53–0.99)*	0.81 (0.51–1.29)	0.87 (0.53–1.43)	0.79 (0.45–1.37)	0.79 (0.44–1.43)

*Abbreviations*: *OR* odds ratio, *CI* confidence interval, *na* not applicable

^a^All indicators are adjusted for sex, age group, socioeconomic index, and ethnic origin

^b^Ethnic origin and religion are closely associated therefore ethnic origin only was used for adjustment

^c^The different water, sanitation and hygiene (WASH)/intervention indicators are closely related with each other therefore separate models adjusting each for sociodemographic indicators and including a random effect coefficient on community level were applied

*Statistically significant with a *P*-value < 0.05

**Statistically significant with a *P*-value < 0.01

Reference categories for explanatories: sex = female, age group = 0–5 years, ethnic origin = allochthonous (all other ethnic groups than the local Baoulé group), religion = Christian, group = control, open defecation free (ODF) status = not odf, toilet ownership = never owner of any latrine in 2011 and 2012, possession of functional latrine in 2012 = no functional latrine in 2012, toilet use in 2012 = household head never used any latrine/toilet in 2012, strict toilet use in 2012 = household head never or irregularly used latrine/toilet for defecation in 2012

In contrast to soil-transmitted helminth infection, WASH indicators and intervention group (OR = 0.68, *P* = 0.015) and ODF community (OR = 0.69, *P* = 0.017) had lower odds for intestinal protozoa infection at the 1-year follow-up. Among the WASH indicators, households with newly constructed latrines (OR = 0.63, *P* = 0.007) showed the highest effect on reduction of intestinal protozoa infection.

### Impact of the intervention on KAPB

Table 5 compares changes in defecation and hygiene behaviour as reported from the household-based questionnaire between control and intervention communities. At baseline, use of latrines and reported hand washing after defecation was significantly lower among households of intervention communities than in control communities (15.5% and 14.9% *vs* 46.7% and 29.7%, respectively). This, however, dramatically changed into the opposite direction after implementation of CLTS (intervention: 94.6% and 41.1%; control: 40.1% and 25.8%). Nearby bushes as the most often stated place for open defecation in 2011 (69.2% and 75.0% in control and intervention communities, respectively) was significantly less often mentioned among households of intervention communities (16.7%, McNemar’s OR = 0.10, *P* <  0.001) in 2012, while it remained constantly high in control communities (64.8%). With regard to children’s defecation behaviour, a similar trend was observed. In 2012, children from households of intervention communities were reported to mainly use latrines/toilets (81.4%), while nearby bushes (64.1%) was still the major place for defecation of children from control communities.

**Table 5:** Comparison of changes in defecation and hygiene behaviour as reported from the household-based questionnaire (*n* = 350) between control and intervention communities

Behaviour	Group	Proportion (%) in 2011	OR (95% CI)	*P*-value	Proportion (%) in 2012	OR (95% CI)	*P*-value	Change between 2011 and 2012 (McNemar’s test)	
								OR (95% CI)	*P*-value
Open defecation (OD)	Control	92.31	1		81.32	1		0.31 (0.13–0.67)	0.002
	Intervention	95.83	1.92 (0.75–4.87)	0.172	44.64	0.19 (0.12–0.30)	< 0.001	0.04 (0.01–0.12)	< 0.001
Place for OD									
Bushes (close to community)	Control	69.23	1		64.84	1		0.71 (0.38–1.31)	0.312
	Intervention	75.00	1.33 (0.83–2.13)	0.230	16.67	0.11 (0.07–0.18)	< 0.001	0.10 (0.05–0.19)	< 0.001
Plantation	Control	65.93	1		71.43	1		1.27 (0.81–2.01)	0.326
	Intervention	48.81	0.49 (0.32–0.76)	0.001	41.07	0.28 (0.18–0.44)	< 0.001	0.73 (0.46–1.15)	0.193
Close to river/pond	Control	4.40	1		0.00	1		0.20 (0.04–1.79)	0.219
	Intervention	2.98	0.67 (0.21–2.08)	0.486	0.60	na	na	na	na
Behind the house	Control	2.75	1		3.30	1		1.25 (0.27–6.30)	1.000
	Intervention	1.19	0.43 (0.82–2.23)	0.312	0.60	0.18 (0.02–1.47)	0.109	0.50 (0.01–9.60)	1.000
Toilet use^a^	Control	46.70	1		40.11	1		0.43 (0.17–0.98)	0.043
	Intervention	15.48	0.21 (0.13–0.35)	< 0.001	94.64	26.38 (12.66–54.96)	< 0.001	134.00 (23.64–5331.72)	< 0.001
Strict toilet use^b^	Control	26.92	1		29.67	1		1.28 (0.66–2.51)	0.533
	Intervention	12.50	0.39 (0.22–0.68)	0.001	79.17	9.01 (5.52–14.70)	< 0.001	17.00 (8.00–43.20)	< 0.001
Spontaneously mentioned to wash hands after defecation	Control	29.67	1		25.82	1		0.83 (0.50–1.34)	0.483
	Intervention	14.88	0.41 (0.24–0.71)	0.001	41.07	2.00 (0.25–3.15)	0.003	3.93 (2.20–7.47)	< 0.001
Place of defecation of households’ children^c^									
Toilet/latrine	Control	26.95	1		26.95	1		1.00 (0.49–2.04)	1.000
	Intervention	8.92	0.27 (0.14–0.51)	< 0.001	81.35	11.97 (7.05–20.30)	< 0.001	58.00 (15.70–484.61)	< 0.001
Bushes (close to community)	Control	62.28	1		64.07	1		1.19 (0.58–2.47)	0.736
	Intervention	78.34	2.19 (1.34–3.58)	0.002	4.52	0.03 (0.01–0.06)	< 0.001	0.17 (0.00–0.06)	< 0.001
Behind the house	Control	13.77	1		14.37	1		1.05 (0.54–2.04)	1.000
	Intervention	1.91	0.12 (0.04–0.42)	0.001	0.00	na	na	0.00 (0.00–2.42)	0.250
Potty	Control	11.38	1		47.31	1		11.00 (4.79–31.05)	< 0.001
	Intervention	19.75	1.92 (1.03–3.56)	0.039	65.97	2.16	0.001	9.34 (4.52–22.51)	< 0.001
On the field/plantation	Control	5.99	1		7.19	1		1.22 (0.46–3.34)	0.824
	Intervention	5.73	0.96 (0.38–2.42)	0.922	0.00	na	na	0.00 (0.00–0.51)	0.003
Close to river/pond	Control	0.00	1		18.56	1		na	na
	Intervention	0.00	na	na	20.13	1.11 (0.64–1.92)	0.722	na	na

^a^Toilet use defined as use of a latrine/toilet (private or shared) of any frequency and unconditional of other places used for defecation

^b^Strict toilet use defined as use of a latrine/toilet (private or shared) of a minimum frequency of 2 (includes “regularly”, “often” and “always”) combined with no open defecation except for defecation in the plantation far away from the community

^c^Only for households with children (*n* = 324)

Not only reported practice with regard to defecation and hygiene changed among households from intervention communities, but also awareness for problems associated with open defecation. Table 6 shows that the ranking changed from aspects of safety and comfort primarily mentioned in 2011 to contamination of the environment and hygiene perceived as main problems associated with open defecation in 2012. The spontaneous reporting of “polluting environment” exclusively significantly increased among households from intervention communities (from 20.4% to 52.2%, McNemar’s OR = 6.56, *P* <  0.001).

**Table 6:** Comparison of changes in awareness and attitudes towards continued open defecation and latrine ownership as reported spontaneously from the household-based questionnaire between control and intervention communities

Awarness and attitude	Group	Proportion (%) in 2011	OR (95% CI)	*P*-value	Proportion (%) in 2012	OR (95% CI)	*P*-value	Change between 2011 and 2012 (McNemar’s test)	
								OR (95% CI)	*P*-value
Problems associated with open defecation (*n* = 315), spontaneously mentioned									
Safety	Control	51.90	1		28.48	1		0.30 (0.16–0.54)	< 0.001
	Intervention	56.05	1.18 (0.76–1.84)	0.460	23.57	0.77 (0.47–1.28)	0.321	0.23 (0.12–0.40)	< 0.001
Hygiene	Control	24.05	1		34.18	1		1.70 (0.99–2.97)	0.056
	Intervention	26.11	1.12 (0.67–1.86)	0.673	36.31	1.10 (0.69–1.74)	0.693	1.55 (0.95–2.57)	0.081
No comfort	Control	22.15	1		6.33	1		0.19 (0.07–0.47)	< 0.001
	Intervention	28.66	1.41 (0.85–2.35)	0.185	8.28	1.34 (0.57–3.14)	0.507	0.22 (0.09–0.46)	< 0.001
Privacy	Control	22.15	1		22.78	1		1.04 (0.57–1.90)	1.000
	Intervention	5.73	0.21 (0.10–0.46)	< 0.001	11.46	0.44 (0.24–0.81)	0.009	2.8 (0.95–9.93)	0.064
Pollutes environment	Control	20.89	1		18.35	1		0.85 (0.47–1.54)	0.672
	Intervention	20.38	0.97 (0.56–1.67)	0.912	52.23	4.86 (2.92–8.10)	< 0.001	6.56 (3.23–15.04)	< 0.001
Difficulties for elderly or sick people	Control	9.49	1		1.90	1		0.14 (0.02–0.62)	0.004
	Intervention	2.55	0.25 (0.08–0.77)	0.016	1.91	1.01 (0.20–5.06)	0.994	0.75 (0.11–4.43)	1.000
Visitors	Control	5.06	1		8.23	1		1.83 (0.62–6.04)	0.332
	Intervention	1.27	0.24 (0.05–1.16)	0.076	5.10	0.60 (0.24–1.49)	0.269	4.00 (0.80–38.67)	0.109
Abundance of flies/insects	Control	2.53	1		20.25	1		8.00 (2.84–31.14)	< 0.001
	Intervention	5.73	2.34 (0.71–7.77)	0.164	18.47	0.89 (0.51–1.56)	0.689	3.86 (1.64–10.49)	< 0.001
Drinking water quality	Control	2.53	1		0.63	1		0.25 (0.01–2.53)	0.375
	Intervention	1.27	0.50 (0.09–2.75)	0.423	0.64	1.01 (0.06–16.23)	0.996	0.50 (0.01–9.61)	1.000
Reasons to possess a latrine (*n* = 89 in 2011, *n* = 249 in 2012, *n* = 69 with data for both years)^a^, spontaneously mentioned									
Comfort	Control	44.12	1		8.22	1		0.05 (0.00–0.30)	< 0.001
	Intervention	33.33	0.63 (0.23–1.77)	0.383	9.09	1.12 (0.42–2.98)	0.825	0.00 (0.00–0.85)	0.031
Clean environment	Control	32.35	1		31.51	1		0.80 (0.27–2.25)	0.815
	Intervention	42.86	1.57 (0.58–4.27)	0.379	46.02	1.85 (1.04–3.30)	0.036	1.00 (0.13–7.47)	1.000
Safety	Control	32.35	1		30.14	1		0.54 (0.18–1.45)	0.263
	Intervention	28.57	0.84 (0.29–2.45)	0.744	18.75	0.53 (0.29–1.00)	0.051	1.00 (0.19–5.37)	1.000
Privacy	Control	20.59	1		23.29	1		0.86 (0.27–2.76)	1.000
	Intervention	19.05	0.91 (0.26–3.13)	0.878	21.59	0.91 (0.47–1.74)	0.769	1.67 (0.32–10.73)	0.727
Avoid diseases	Control	19.12	1		38.36	1		2.50 (0.92–7.86)	0.078
	Intervention	14.29	0.71 (0.18–2.76)	0.616	39.77	1.06 (0.61–1.86)	0.835	5.00 (0.56–236.49)	0.219
Easier for elderly or sick people	Control	17.65	1		10.96	1		0.38 (0.06–1.56)	0.227
	Intervention	0.00	na	na	1.70	0.14 (0.04–0.55)	0.005	na	
Hygiene/Health	Control	8.82	1		39.73	1		16.00 (2.49–670.96)	< 0.001
	Intervention	19.05	2.43 (0.62–9.61)	0.205	28.41	0.60 (0.34–1.07)	0.082	2.50 (0.41–26.25)	0.453
Visitors	Control	8.82	1		13.70	1		1.40 (0.38–5.59)	0.774
	Intervention	0.00	na	na	9.09	0.63 (0.27–1.46)	0.282	na	na
Reason to practice open defecation (*n* = 349 for 2011, *n* = 188 for 2012, *n* = 149 with data for both years)^a^, spontaneously mentioned									
No functional latrine on spot	Control	77.71	1		72.79	1		0.91 (0.46–1.77)	0.875
	Intervention	90.10	2.61 (1.43–4.78)	0.002	41.46	0.27 (0.13–0.54)	< 0.001	na	< 0.001
Traditional habit	Control	22.93	1		10.88	1		0.25 (0.08–0.63)	0.001
	Intervention	18.23	0.75 (0.44–1.26)	0.279	7.32	0.65 (0.18–2.34)	0.506	0.17 (0.02–0.75)	0.013
Comfort	Control	4.46	1		2.04	1		0.40 (0.04–2.44)	0.453
	Intervention	2.60	0.57 (0.18–1.84)	0.350	0.00	na	na	na	na
Difficulties with maintenance/construction	Control	0.64	1		5.44	1		5.00 (0.56–236.49)	0.219
	Intervention	0.00	na	na	9.76	1.88 (0.54–6.58)	0.324	na	

^a^Selective question type; total number of respondents therefore changed by year and only households who did not undergo change in toilet ownership or practice of open defecation could be analysed for changes over time, as assessed by McNemar’s test

## Discussion

Our study presents new evidence on potential gains of an integrated control approach consisting of preventive chemotherapy, CLTS and health education against helminthiases and intestinal protozoa infection in a rural setting of south-central Côte d’Ivoire. Over the past decade, CLTS as a means to prevent diarrhoeal diseases has gained traction and is being applied at large scale in various low- and middle-income countries [22, 35]. Yet, there is relatively little scientific inquiry about the specific effects of CLTS alone or in combination with other interventions against neglected tropical diseases [13, 36–39], and hence, the public health impact of CLTS [40]. We discuss the effect of an integrated package of interventions, placing emphasis on latrine construction by the community, levels of helminth and intestinal protozoa infections, infection patterns at follow-up and people’s behaviour and attitudes (e.g. latrine use *vs* continued open defecation).

### Community response and latrine construction

Our package of interventions resulted in a strong response in the five selected communities. Indeed, four out of the five communities were declared ODF within six months, whilst in the fifth community more than 80% of the households had latrines (Fig. 3). Particularly small communities (e.g. Amani Kouadiokro and Kouadio Kouamékro) rapidly reached 100% latrine coverage, and hence, were certified ODF. This corroborates recent findings from a CLTS programme conducted in Ghana that found the largest impact in small, remote villages with low exposure to prior water and sanitation projects [41]. Hamlets and small villages have the advantage to be usually socially cohesive with a more clear and respected hierarchy that may result in a faster and higher degree of social mobilisation of the associated community members. In larger villages, such as Katchénou where 17 weeks after triggering still fewer than 10% of the households possessed a latrine, conducting a health education campaign showed an impressive boosting effect. This underlines the need for integrated approaches combining CLTS or other sanitation programmes with health education. Future interventions in large communities, however, may represent a challenge for community mobilisation and need to address the role of communication and leadership (e.g. by repeated triggering, choice of “good” religious and/or secular leaders and community facilitators) [21, 42]. Compared to other CLTS programmes conducted in Eastern Africa, we reached ODF status within a relatively short period of time. Crocker et al. [43] explain low changes in open-defection reduction and increase in latrine ownership with generally low open-defecation in Ethiopia.

### Impact on helminth and intestinal protozoa infection

Interestingly, we did not observe significant differences in soil-transmitted helminth reinfection rates when comparing intervention and control communities. This may be partly explained by still relatively low reinfection rates in both groups (control: 7.0%, intervention: 11.8%), perhaps indicating a too short follow-up period (in our case five months). Our assumption was that infection levels in the control communities after a single round of preventive chemotherapy would have approached baseline levels (here > 25%) within about a year [3]. Impact assessment for helminth infection was further hampered by significant discrepancies in baseline infection levels between groups. While soil-transmitted helminth infections were more prevalent among intervention communities, schistosomiasis showed a highly focally distribution, as it mainly occurred in a single locality that was part of the control group. It should be noted, however, that a negative relationship between hookworm infections and toilet ownership and use was evident in the baseline data of the same study cohort [27]. Further, the finding on a significant difference in the extent of proportion of decrease in hookworm infection indicates that baseline positives that became negative were also more likely to remain negative five months after treatment within the intervention group (Table 3).

For intestinal protozoa, although not tackled through the administration of anthelminthic drugs as part of the preventive chemotherapy component, we found significantly lower infection rates in the intervention communities at follow-up, while infection levels in control communities remained unchanged (Table 2). Baseline uninfected individuals were further found to be less likely to become newly infected with *E. nana* or *Blastocystis* sp. if they belonged to the intervention group as highlighted by a statistically significantly lower proportion of increase (Table 3). In the multivariable logistic regression analysis, the following variables were associated with significantly lower odds for intestinal protozoa infections: intervention community, ODF status, ownership and use of toilet. Lower intestinal protozoa infection levels and lower hookworm egg counts in intervention communities may serve as biological indicators for decreased environmental faecal pollution [44] and reduced abundance of flies acting as potential carriers [45]. Hence, our findings strengthen the assumption of a direct impact from the intervention package.

Other studies on community level incorporating a sanitation package to fight enteric parasite infections showed mixed results. Studies that reached high latrine coverage and with long-term follow-up [10] resulted in higher impact on prevalence and incidence, compared to studies achieving low latrine coverage [16] and high levels of continued open defecation [15].

### Potential factors guiding infection status at follow-up

Reinfection patterns for soil-transmitted helminths highlight potential sources for continued transmission despite ODF status in intervention communities. School-aged children were at the highest risk of reinfection (23 out of 50 new infections in the intervention communities), which might, at least partly, be explained by children attending the nearest school that might be located in another community not yet declared ODF. Such reinfections might be considered as imported infections, dampening the impact for soil-transmitted helminthiasis control in intervention communities. Another potential source for reinfection could be seen in plantation sites, where open defecation is continued, as latrines were only built in close proximity to the households. In intervention communities, reported open defecation while people pursue agricultural activities remained relatively high (41.1%). Logistic regression modelling indicated potential cultural difference in hygiene practices, as expressed by a higher risk for intestinal protozoa (re)infections among the autochthonous population and reduced risk among Muslim community members. In previous research, males have been found to be more prone for reinfection, as they are less adhering to good defecation practices and hygiene behaviour [9, 46, 47]. In our study, however, this gender-related reinfection pattern was no longer observed in the intervention communities compared to control communities where it persisted. How much of this can be related to positive change in defecation and hygiene behaviour among males and to what extent the interventions triggered it remains to be clarified.

### Impact on behaviour and attitudes

Apart from access to sanitation facilities, attitudes towards defecation and hygiene practices, acceptance and (appropriate) use of these infrastructures are key determinants for discontinuation of open defecation and thus successful interruption of faecal contamination of the environment [15, 16]. Our findings showed high proportion of behaviour change within the intervention communities with considerably reduced reported open defecation in general (before: 95.8%, after: 44.6%), going hand-in-hand with an increase in stated toilet use (before: 15.5%, after: 94.6%). Especially open-defecation in close proximity to the households, which probably serves as the main source for infections and transmission via flies, plummeted in both adults (before: 75.0%, after: 16.7%) and children (before: 78.3%, after: 4.5%). Similarly to findings from a CLTS trial in rural Mali [13], household heads from intervention communities significantly more often reported washing hands after defecating. The intervention significantly increased perceptions of open defecation as polluting the environment among beneficiary communities. This may be explained by the emphasis given to activities focusing on projection of the extent of environmental contamination through faeces during CLTS triggering (e.g. mapping, calculating “caca”, transect walk and identifying the dirtiest neighbourhoods) [22].

Nevertheless, it is difficult to say to what extent CLTS alone or the combination of health education and preventive chemotherapy improved people’s behaviour of defecation and general hygiene and cleanliness. Lawrence et al. [48] identified shame from the CLTS triggering as one of the most important factors for behavioural change in rural Zambia. Yet, CLTS may also influence adherence through other social and emotive factors that include disgust, pressure from hierarchical powers and community groups, and competition among villages to achieve ODF status. Health education complements by addressing lacking knowledge on disease transmission and prevention. Raising awareness is particularly important where local concepts of helminthiases and diarrhoeal diseases may foster risk-related behaviour or reduce adherence to best practices [49]. Further research on factors improving adherence to CLTS and health education interventions is warranted and may contribute to enhancing community effectiveness of such interventions.

### Strengths and limitations

A strength of our study is the rigorous sanitation approach, namely CLTS, which implies a very high private latrine ownership coverage and, ideally, the certification of ODF status in an intervention community. Furthermore, adherence may be enhanced due to non-subsidized latrine provision [16, 50, 51]. However, our assessment on behaviour change and adherence to hygiene practices is based on reported frequencies from cross-sectional questionnaire surveys administered to household heads and is not supported by direct observations. Thus, the assumption of the link between access to a private latrine and its use might not be entirely valid.

Certain considerations with regard to study design should be taken into account in future studies to generate stronger evidence. Specifically, inclusion of more localities is pivotal, so that bias from local heterogeneity of helminth and intestinal protozoa infection can be mitigated. Furthermore, a cluster randomised controlled trial design generates stronger evidence, since potential baseline differences in disease prevalence, population size, level of existing water and sanitation infrastructure are addressed through randomisation [7]. Sustainability of CLTS is a critical issue [36] and remains to be assessed in different social-ecological settings. Our study had a relatively short follow-up period between the last round of anthelminthic treatment and the follow-up parasitological assessments and thus might not have allowed measuring the full impact on helminth infections. A prolonged survey period would thus improve surveillance data with regard to reinfection patterns as well as create new evidence on long-term effects of CLTS and health education as a means for controlling and eliminating neglected tropical diseases [10].

## Conclusions

CLTS in combination with health education and preventive chemotherapy holds promise as an effective participatory approach that can be applied at community level to improve people’s health and wellbeing. In our view, the results obtained here from a primarily rural setting of Côte d’Ivoire with high levels of open-defecation and low access to sanitation programmes at the start of the interventions are relevant for other settings in low- and middle-income countries [13]. CLTS implemented at a large scale can further contribute to reach the sixth of the defined Sustainable Development Goals, namely to ensure availability and sustainable management of water and sanitation for all [52]. To assess potential gains in neglected tropical diseases control from such an integrated approach compared to preventive chemotherapy alone and to evaluate its sustainability, additional research is warranted, using rigorous study designs, such as cluster-randomised trials.

## Additional files

Additional file 1: Table S1. Characteristics of the final cohort. (DOCX 14 kb)

Additional file 2: Table S2. Mean infection intensity in control and intervention communities during baseline and follow-up surveys. (DOCX 17 kb)

## Abbreviations

CI: Confidence interval
CLTS: Community-led total sanitation
CSRS: Centre Suisse de Recherches Scientifiques en Côte d’Ivoire
DALY: Disability-adjusted life year
epg: Eggs per gram of stool
ERR: Egg reduction rate
HDSS: Health and demographic surveillance system
KAPB: Knowledge, attitudes, practices and beliefs
NGO: Non-governmental organisation
ODF: Open defecation free
OR: Odds ratio
SAF: Sodium acetate-acetic acid-formalin
UNICEF: United Nations Children’s Fund
WASH: Water, sanitation and hygiene
WHO: World Health Organization

## References

[1] GBD 2015 DALYs and HALE Collaborators. Global, regional, and national disability-adjusted life-years (DALYs) for 315 diseases and injuries and healthy life expectancy (HALE), 1990-2015: a systematic analysis for the global burden of disease study 2015. Lancet. 2016;388(10053):1603–58. 10.1016/S0140-6736(16)31460-X PMC5388857 27733283

[2] WHO. Integrating neglected tropical diseases in global health and development: fourth WHO report on neglected tropical diseases. Geneva: World Health Organization; 2017.

[3] Jia TW, Melville S, Utzinger J, King CH, Zhou XN (2012). Soil-transmitted helminth reinfection after drug treatment: a systematic review and meta-analysis. PLoS Negl Trop Dis.

[4] Prüss-Üstün A, Bos R, Gore F, Bartram J. Safe water, better health: cost, benefits and sustainability of interventions to protect and promothe health. Geneva: World Health Organization; 2008.

[5] Grimes JET, Croll D, Harrison WE, Utzinger J, Freeman MC, Templeton MR. The relationship between water, sanitation and schistosomiasis: a systematic review and meta-analysis. PLoS Negl Trop Dis. 2014;8(12):e3296. 10.1371/journal.pntd.0003296 PMC4256273 25474705

[6] Strunz EC, Addiss DG, Stocks ME, Ogden S, Utzinger J, Freeman MC (2014). Water, sanitation, hygiene, and soil-transmitted helminth infection: a systematic review and meta-analysis. PLoS Med.

[7] Cairncross S, Hunt C, Boisson S, Bostoen K, Curtis V, Fung IC, Schmidt WP (2010). Water, sanitation and hygiene for the prevention of diarrhoea. Int J Epidemiol.

[8] Wolf J, Prüss-Ustün A, Cumming O, Bartram J, Bonjour S, Cairncross S, et al. Assessing the impact of drinking water and sanitation on diarrhoeal disease in low- and middle-income settings: systematic review and meta-regression. Trop Med Int Health. 2014;19(8):928–42. 10.1111/tmi.12331 24811732

[9] Freeman MC, Clasen T, Brooker SJ, Akoko DO, Rheingans R (2013). The impact of a school-based hygiene, water quality and sanitation intervention on soil-transmitted helminth reinfection: a cluster-randomized trial. Am J Trop Med Hyg.

[10] Steinmann P, Yap P, Utzinger J, Du ZW, Jiang JY, Chen R (2015). Control of soil-transmitted helminthiasis in Yunnan province, People's Republic of China: experiences and lessons from a 5-year multi-intervention trial. Acta Trop.

[11] Garn JV, Brumback BA, Drews-Botsch CD, Lash TL, Kramer MR, Freeman MC (2016). Estimating the effect of school water, sanitation, and hygiene improvements on pupil health outcomes. Epidemiology.

[12] Trinies V, Garn JV, Chang HH, Freeman MC (2016). The impact of a school-based water, sanitation, and hygiene program on absenteeism, diarrhea, and respiratory infection: a matched-control trial in Mali. Am J Trop Med Hyg.

[13] Pickering AJ, Djebbari H, Lopez C, Coulibaly M, Alzua ML (2015). Effect of a community-led sanitation intervention on child diarrhoea and child growth in rural Mali: a cluster-randomised controlled trial. Lancet Glob Health.

[14] Freeman MC, Clasen T, Dreibelbis R, Saboori S, Greene LE, Brumback B (2014). The impact of a school-based water supply and treatment, hygiene, and sanitation programme on pupil diarrhoea: a cluster-randomized trial. Epidemiol Infect.

[15] Patil SR, Arnold BF, Salvatore AL, Briceno B, Ganguly S, Colford JM, Gertler PJ (2014). The effect of India's total sanitation campaign on defecation behaviors and child health in rural Madhya Pradesh: a cluster randomized controlled trial. PLoS Med.

[16] Clasen T, Boisson S, Routray P, Torondel B, Bell M, Cumming O (2014). Effectiveness of a rural sanitation programme on diarrhoea, soil-transmitted helminth infection, and child malnutrition in Odisha, India: a cluster-randomised trial. Lancet Glob Health.

[17] Sclar GD, Penakalapati G, Amato HK, Garn JV, Alexander K, Freeman MC (2016). Assessing the impact of sanitation on indicators of fecal exposure along principal transmission pathways: a systematic review. Int J Hyg Environ Health.

[18] Hotez PJ, Kamath A. Neglected tropical diseases in sub-Saharan Africa: review of their prevalence, distribution, and disease burden. PLoS Negl Trop Dis. 2009;3(8):e412. 10.1371/journal.pntd.0000412 PMC2727001 19707588

[19] Utzinger J, Raso G, Brooker S, de Savigny D, Tanner M, Ornbjerg N (2009). Schistosomiasis and neglected tropical diseases: towards integrated and sustainable control and a word of caution. Parasitology.

[20] UN. The millennium development goals. Report 2010. Geneva: World Health Organization; 2010.

[21] Kar K, Chambers R (2008). Handbook on community-led total sanitation.

[22] Bongartz P, Musyoki SM, Milligan A, Holly A (2010). Tales of shit: community-led total sanitation in Africa.

[23] Chambers R (1994). The origins and practice of participatory rural appraisal. World Dev.

[24] Jenkins MW, Curtis V (2005). Achieving the 'good life': why some people want latrines in rural Benin. Soc Sci Med.

[25] Jenkins MW, Scott B (2007). Behavioral indicators of household decision-making and demand for sanitation and potential gains from social marketing in Ghana. Soc Sci Med.

[26] Koné S, Baikoro N, N'Guessan Y, Jaeger FN, Silué KD, Fürst T (2015). Health & demographic surveillance system profile: the Taabo health and demographic surveillance system, Côte d'Ivoire. Int J Epidemiol.

[27] Schmidlin T, Hürlimann E, Silué KD, Yapi RB, Houngbedji C, Kouadio BA, et al. Effects of hygiene and defecation behavior on helminths and intestinal protozoa infections in Taabo, Côte d'Ivoire. PLoS One. 2013;8(6):e65722. 10.1371/journal.pone.0065722 PMC3688730 23840358

[28] Katz N, Chaves A, Pellegrino JA. Simple device for quantitative stool thick-smear technique in schistosomiasis mansoni. Rev Inst Med Trop São Paulo. 1972;14(6):397–400. 4675644

[29] Montresor A, Crompton DWT, Bundy DAP, Hall A, Savioli L (1998). Guidelines for the evaluation of soil-transmitted helminthiasis and schistosomiasis at community level: a guide for control managers.

[30] Savioli L, Hatz C, Dixon H, Kisumku UM, Mott KE. Control of morbidity due to *Schistosoma haematobium* on Pemba Island: egg excretion and hematuria as indicators of infection. Am J Trop Med Hyg. 1990;43(3):289–95. 10.4269/ajtmh.1990.43.289 2121056

[31] Utzinger J, Botero-Kleiven S, Castelli F, Chiodini PL, Edwards H, Kohler N (2010). Microscopic diagnosis of sodium acetate-acetic acid-formalin-fixed stool samples for helminths and intestinal protozoa: a comparison among European reference laboratories. Clin Microbiol Infect.

[32] Kar K, Pasteur K. Subsidy or self-respect? Community-led total sanitation. An update on recent developments. Brighton: Institute of Development Studies; 2005.

[33] Fleiss JL (1981). Statistical methods for rates and proportions.

[34] Twisk JWR (2003). Applied longitudinal data analysis for epidemiology. A practical guide.

[35] Singeling M (2016). To ODF and beyond: sharing experience from the Pan African CLTS Programme.

[36] Belizario VY Jr, Liwanag HJ, Naig JR, Chua PL, Madamba MI, Dahildahil RO. Parasitological and nutritional status of school-age and preschool-age children in four villages in southern Leyte, Philippines: lessons for monitoring the outcome of community-led total sanitation. Acta Trop. 2015;141(Pt A):16–24. 10.1016/j.actatropica.2014.09.008 25255966

[37] Jung S, Doh YA, Bizuneh DB, Beyene H, Seong J, Kwon H (2016). The effects of improved sanitation on diarrheal prevalence, incidence, and duration in children under five in the SNNPR state, Ethiopia: study protocol for a randomized controlled trial. Trials.

[38] Njuguna J (2016). Effect of eliminating open defecation on diarrhoeal morbidity: an ecological study of Nyando and Nambale sub-counties, Kenya. BMC Public Health.

[39] Zimba R, Ngulube V, Lukama C, Manangi A, Tiwari A, Osbert N (2016). Chiengi district, Zambia open defecation free after 1 year of community-led total sanitation. Am J Trop Med Hyg.

[40] Mills JE, Cumming O (2016). The impact of water, sanitation and hygiene on key health and social outcomes: review of evidence.

[41] Crocker J, Abodoo E, Asamani D, Domapielle W, Gyapong B, Bartram J (2016). Impact evaluation of training natural leaders during a community-led total sanitation intervention: a cluster-randomized field trial in Ghana. Environ Sci Technol.

[42] Alzua ML, Cardenas JC, Djebbari H (2014). Community mobilization around social dilemmas: evidence from lab experiments in rural Mali.

[43] Crocker J, Geremew A, Atalie F, Yetie M, Bartram J (2016). Teachers and sanitation promotion: an assessment of community-led total sanitation in Ethiopia. Environ Sci Technol.

[44] Gamboa MI, Basualdo JA, Cordoba MA, Pezzani BC, Minvielle MC, Lahitte HB (2003). Distribution of intestinal parasitoses in relation to environmental and sociocultural parameters in La Plata, Argentina. J Helminthol.

[45] Getachew S, Gebre-Michael T, Erko B, Balkew M, Medhin G (2007). Non-biting cyclorrhaphan flies (Diptera) as carriers of intestinal human parasites in slum areas of Addis Ababa, Ethiopia. Acta Trop.

[46] Kumar R, Rouhani S, Gautam A, Das M (2015). Improving sanitation and hygiene practices of the rural poor through community institutions in Uttar Pradesh, India.

[47] Garn JV, Mwandawiro CS, Nikolay B, Drews-Botsch CD, Kihara JH, Brooker SJ, et al. *Ascaris lumbricoides* infection following school-based deworming in western Kenya: assessing the role of pupils' school and home water, sanitation, and hygiene exposures. Am J Trop Med Hyg. 2016;94(5):1045–54. 10.4269/ajtmh.15-0362 PMC4856601 26903608

[48] Lawrence JJ, Yeboah-Antwi K, Biemba G, Ram PK, Osbert N, Sabin LL, Hamer DH (2016). Beliefs, behaviors, and perceptions of community-led total sanitation and their relation to improved sanitation in rural Zambia. Am J Trop Med Hyg.

[49] Acka CA, Raso G, N'Goran EK, Tschannen AB, Bogoch II, Seraphin E, et al. Parasitic worms: knowledge, attitudes, and practices in western Côte d'Ivoire with implications for integrated control. PLoS Negl Trop Dis. 2010;4(12):e910. 10.1371/journal.pntd.0000910 PMC3006135 21200423

[50] Hueso A, Bell B. An untold story of policy failure: the total sanitation campaign in India. Water Policy. 2013;15(6):1001–17.

[51] Caruso BA, Dreibelbis R, Ogutu EA, Rheingans R (2014). If you build it will they come? Factors influencing rural primary pupils' urination and defecation practices at school in western Kenya. J Water Sanit Hyg Dev.

[52] United Nations: Sustainable development goal 6. [http://www.un.org/sustainabledevelopment/water-and-sanitation/]. Accessed 1 March 2017.

