# Supplementary material for: Effect of an integrated intervention package of preventive chemotherapy, community-led total sanitation and health education on the prevalence of helminth and intestinal protozoa infections in Côte d’Ivoire
Source: Parasit Vectors. 2018 Feb 27;11:115. doi: 10.1186/s13071-018-2642-x (PMC6389068; doi:10.1186/s13071-018-2642-x)
Supplement: Supplementary file 1 — Characteristics of the final cohort. (DOCX 14 kb) [file 13071_2018_2642_MOESM1_ESM.docx]

**Table S1.** Characteristics of the final cohort.

| **Age group (yrs)** | **Total (%)** | **Male (%)** | **Female (%)** |
| --- | --- | --- | --- |
| <6 | 186 (22.96) | 105 (25.12) | 81 (20.66) |
| 6-15 | 253 (31.23) | 140 (33.49) | 113 (28.83) |
| 16-29 | 106 (13.09) | 43 (10.29) | 63 (16.07) |
| 30-45 | 166 (20.49) | 76 (18.18) | 90 (22.96) |
| >45 | 99 (12.22) | 54 (12.92) | 45 (11.48) |
| Total | 810 (100) | 418 (100) | 392 (100) |
