# Supplementary material for: Effect of an integrated intervention package of preventive chemotherapy, community-led total sanitation and health education on the prevalence of helminth and intestinal protozoa infections in Côte d’Ivoire
Source: Parasit Vectors. 2018 Feb 27;11:115. doi: 10.1186/s13071-018-2642-x (PMC6389068; doi:10.1186/s13071-018-2642-x)
Supplement: Supplementary file 2 — Mean infection intensity in control and intervention communities during baseline and follow-up surveys. (DOCX 17 kb) [file 13071_2018_2642_MOESM2_ESM.docx]

**Table S2.** Mean infection intensity in control and intervention communities during baseline and follow-up surveys.

| Parasite | **Group** | **Baseline positive (n)** | **Baseline**  **GM-EPG** | **IRR (95% CI)** | **P-value** | **Follow-up positive (n)** | **Follow-up**  **GM-EPG** | **IRR (95% CI)** | **P-value** | **ERR (%)** |
| --- | --- | --- | --- | --- | --- | --- | --- | --- | --- | --- |
| *Schistosoma mansoni* | Control | 4 | 20 | 1 |  | 4 | 21 | 1 |  | -6.48 |
|  | Intervention | 4 | 35 | 1.17 (1.00, 1.37) | 0.047 | 3 | 37 | 0.93 (0.80, 1.09) | 0.368 | -6.67 |
| *S. haematobium* | Control | 57 | 14 | 1 |  | 18 | 4 | 1 |  | 69.33 |
|  | Intervention | 0 | 0 | 0.11 (0.09, 0.13) | <0.001 | 3 | 7 | 0.80 (0.69, 0.92) | 0.002 | N/A |
| Hookworm | Control | 97 | 80 | 1 |  | 27 | 68 | 1 |  | 15.23 |
|  | Intervention | 155 | 157 | 3.74 (2.80, 5.00) | <0.001 | 45 | 55 | 0.91 (0.71, 1.18) | 0.488 | 64.88 |
| *Ascaris lumbricoides* | Control | 0 | 0 | N/A |  | 0 | 0 | N/A |  | N/A |
|  | Intervention | 3 | 435 | N/A |  | 1 | 2232 | N/A |  | -413.13 |
| *Trichuris trichiura* | Control | 5 | 155 | 1 |  | 0 | 0 | N/A |  | 100.00 |
|  | Intervention | 11 | 201 | 6.07 (4.71, 7.83) | <0.001 | 6 | 26 | N/A |  | 86.96 |

Negative binomial regressions were used to derive incidence ratio rates for comparison of egg counts of control and intervention communities at baseline and follow-up. The egg reduction rate was calculated as [1 – (GM-EPG at follow-up/ GM-EPG at baseline)] *100 for control and intervention communities separately.

GM-EPG=Geometric mean egg count (eggs per gram)

IRR=incidence rate ratio

CI=confidence interval

ERR=Egg reduction rate

N/A=not applicable
